# Application of droplet digital PCR for the detection of vector copy number in clinical CAR/TCR T cell products

**DOI:** 10.1186/s12967-020-02358-0

**Published:** 2020-05-08

**Authors:** Alex Lu, Hui Liu, Rongye Shi, Yihua Cai, Jinxia Ma, Lipei Shao, Victor Rong, Nikolaos Gkitsas, Hong Lei, Steven L. Highfill, Sandhya Panch, David F. Stroncek, Ping Jin

**Affiliations:** Center for Cellular Engineering, Department of Transfusion Medicine and Cellular Engineering, NIH Clinical Center, Bethesda, MD USA

**Keywords:** Droplet digital PCR, Vector copy number, Genetically engineered T cells, Gene therapy, Chimeric antigen receptor (CAR) T cells, T Cell Receptor (TCR)-engineered T cells, Cellular cancer immunotherapy

## Abstract

**Background:**

Genetically engineered T cells have become an important therapy for B-cell malignancies. Measuring the efficiency of vector integration into the T cell genome is important for assessing the potency and safety of these cancer immunotherapies.

**Methods:**

A digital droplet polymerase chain reaction (ddPCR) assay was developed and evaluated for assessing the average number of lenti- and retroviral vectors integrated into Chimeric Antigen Receptor (CAR) and T Cell Receptor (TCR)-engineered T cells.

**Results:**

The ddPCR assay consistently measured the concentration of an empty vector in solution and the average number of CAR and TCR vectors integrated into T cell populations. There was a linear relationship between the average vector copy number per cell measured by ddPCR and the proportion of cells transduced as measured by flow cytometry. Similar vector copy number measurements were obtained by different staff using the ddPCR assay, highlighting the assays reproducibility among technicians. Analysis of fresh and cryopreserved CAR T and TCR engineered T cells yielded similar results.

**Conclusions:**

ddPCR is a robust tool for accurate quantitation of average vector copy number in CAR and TCR engineered T cells. The assay is also applicable to other types of genetically engineered cells including Natural Killer cells and hematopoietic stem cells.

## Background

Chimeric antigen receptor (CAR) T cells are a novel cellular therapy wherein autologous T cells are harvested from a patient and genetically modified to express chimeric antigen receptors. These receptors are composed of an extracellular scFv designed to bind to a target with high specificity, and an intracellular portion consisting of a costimulatory structure and the T Cell Receptor (TCR) zeta chain which activates T cells [1, 2]. The purpose of these modifications is to generate a T cell that will, with high specificity, activate and expand in vivo when exposed to a target molecule. A common application of CAR T cells is in treating B cell malignancies which overexpress extracellular markers [1–6]. T cells genetically engineered to express TCRs specific for cancer antigens are also being used for cancer immunotherapy. T cells engineered to express TCRs directed to the cancer/testis antigen NY-ESO-1 are being used to treat melanoma and synovial cell sarcoma [7]. Recently, TCR engineered T cells directed to Human Papilloma Virus (HPV) 16 E6 oncoprotein have been used treat HPV-associated epithelial cancers [8].

The transgenes coding for CAR and TCR are most often introduced using replication incompetent retroviral or lentiviral vectors, a process which integrates transgenes into the T cell genome and carries a degree of risk. The number of copies of transgenes that have integrated into the cells’ genome has been associated with the clinical potency of these genetically engineered T cells. It is desirable to attain a high enough average copy number in a given cell population for a product to be effective. However, greater transgene copy numbers are also associated with a higher risk of genotoxicity with higher probability of transgene integration near oncogenes. Subsequently, safety testing of genetically engineered T cell products is paramount with particular attention paid to keeping transgene copy numbers within a safe but effective range [9].

Droplet digital PCR (ddPCR) has emerged as a recent technology which allows for more precise quantification and analysis of DNA and RNA as compared to older PCR techniques such as real-time PCR [10]. Applications of ddPCR include assessment of gene copy number variation, vector titer and rare event detection [11–13]. It has been used to measure the number of copies of retroviral vectors integrated into induced pluripotent stem (iPS) cells [14] and CD34+ hematopoietic stem cells [15]. It has also been used to assess the number of integrated copies of lentiviral [16] and adenoviral vectors [13].

The primary innovations in ddPCR are the partitioning of samples into thousands of nano-liter sized droplets and the binary evaluation of fluorescence in each of these droplets. Two fluorescent molecules are used in an assay: a reference probe, which binds to a reporter gene present in all cells being assayed, and a vector probe, which binds to the sequence of the vector being evaluated for copy number. Each of the droplets generated in ddPCR serves as an individual chamber for product amplification and they are analyzed individually after the reaction as either positive or negative for the presence of the two fluorescent targeting molecules. The ratio of droplets positive for both the reference and vector probe versus droplets positive for the reference probe is representative of the amount of vector copies present in the overall population. The assumption of the Poisson distribution then allows for calculation of the average number of copies of the vector present in the cell population. Notably this removes the need to generate a standard curve as fluorescence is measured in a binary rather than relative fashion which lends to the precision of ddPCR. Comparisons of the precision in absolute quantitation using ddPCR versus real-time PCR have shown ddPCR to be significantly more precise, with up to a seven fold reduction in variation of measurements [17].

ddPCR also holds value in its ability to accurately detect copy numbers with a wide dynamic range. For example, determining the presence and copy number of oncogenes in a cancer sample can help with diagnosis and treatment. Linear regression analysis was performed on data sets generated by fluorescence in situ hybridization (FISH) and ddPCR for the MET gene copy number in cancer samples and found a high correlation between the data produced by the two techniques [18]. Another analysis compared ddPCR with qPCR and Southern blot techniques for the determination of copy number of transgenes in sugar cane with the conclusion that ddPCR had superior accuracy [19]. ddPCR was also used in analyzing transgene copy number in induced pluripotent stem cells for applications in gene therapy and it was found to be superior to qPCR [13]. Having demonstrated both accuracy and precision when validated against other methods of determining copy number, ddPCR has clear potential in diagnostics and genetic engineering.

To that end, we validated ddPCR as a means of screening genetically engineered CAR and TCR T cell products for transgene copy number. Aspects of the ddPCR validation included assessing its accuracy across different copy number levels, along with its precision across time points, across technicians, and across labs. Additionally, preliminary experiments were performed using ddPCR to explore the impact of different manufacturing parameters on resulting transgene copy number. These results demonstrated the potential application of ddPCR for the measurement of vector copy number in cellular therapies.

## Materials and methods

### Genetically engineered T cell production

The CAR T cell production method is detailed in [20, 21]. In brief, autologous peripheral blood mononuclear cells (PBMCs) collected by apheresis were cultured with an anti-CD3 monoclonal antibody and IL-2 to induce T cell proliferation. The cells were then transduced with a γ-retroviral vector that encoded a CAR and 7 days after initiation of the cultures the CAR T cells were collected for vector copy number analysis. A similar method was used to manufacture TCR engineered T cells [8, 22] using a γ-retroviral vector that encoded a TCR recognizing HPV16 E7 oncoprotein.

### DNA Isolation and Qualification

Genomic DNA was extracted from genetically engineered T cell samples and untransduced control cells using a Qiagen DNeasy Blood and Tissue Kit (Qiagen). The purity and concentration of the DNA samples were measured using a Nanodrop spectrophotometer (Thermo Fisher Scientific).

### ddPCR Copy Number Assays

A Bio-Rad laboratories Auto DG QX200™ ddPCR system was used for all ddPCR experiments analyzing extracted DNA for vector copy numbers. Full protocols are detailed in manufacturers’ materials: Bio-Rad Laboratories, Inc. (2019) *ddPCR™ Supermix for Residual DNA Quantification.* Retrieved from http://www.bio-rad.com/webroot/web/pdf/lsr/literature/10048259.pdf.

Bio-Rad Laboratories, Inc. (2019) *Automated Droplet Generator Instruction Manual.* Retrieved from http://www.bio-rad.com/webroot/web/pdf/lsr/literature/10043138.pdf.

Bio-Rad Laboratories, Inc. (2019) *QX200™ Droplet Reader and QuantaSoft™ Software Instruction Manual.* Retrieved from www.biorad.com/webroot/web/pdf/lsr/literature/10031906.pdf.

### Estimating ddPCR‐based limit of detection

We used an empty lentiviral vector, VSVG, to create 1:5 standard serial dilutions at nine dilution points between 50,000.00 to 0.13 molecules/µL. The 1:5 dilution series was created fresh each time and assessed on droplet digital PCR (ddPCR). We performed ddPCR amplification with the QX200 Droplet Digital PCR System (BioRad, Hercules, CA, USA) [23].

### Flow cytometry analysis

The cells were analyzed using fluorochrome-labeled monoclonal antibodies (mAbs) including anti-human CD3 (clone SK7, BD Biosciences), anti-human TCR beta (clone H57-597, Thermo Fisher Scientific) and anti-human EGFR (clone AY13, BioLegend) as well as viability dye 7-AAD (BD Biosciences). Samples were stained and acquired with BD FACSCanto (BD Biosciences, 2350 Qume Drive, San Jose, CA). Data were analyzed with BD FACSDivaTM software and FlowJo software (BD Biosciences, 2350 Qume Drive, San Jose, CA).

## Results

### Upper and lower limits of detection

**Fig. 1:**
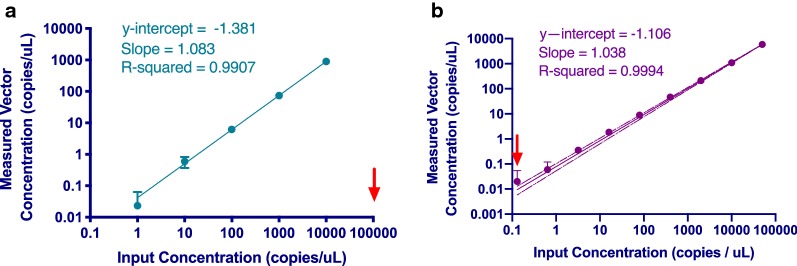
Analysis of Assay Upper and Lower Limits of Detection Using VSVG Vector. Serial dilutions of an empty VSVG viral vector were analyzed using ddPCR and the upper limit of assay was determined to be 1 × 10^4^ copies per microliter and the lower limit was 0.13 copies per microliter. **a** The vector concentrations are indicated on the X-axis and ranged from $1$ to $1 \times 10^{4}$ copies per microliter. The measured vector copy number is shown on the Y- axis. The number of copies input for each resulting observation is shown on the X-axis. Axes are in log–log scale. The line of best fit displayed is a log–log line with a slope of 1.083, a y-intercept of − 1.381 and an R^2^ value of 0.9907. For inputs of $1 \times 10^{5}$ copies per microliter or higher, the Quantasoft software failed to return a measured value. **b** The vector concentrations are indicated on the X-axis and ranged from $0.13$ to $5 \times 10^{4}$ copies per microliter. The measured vector copy number is shown on the Y- axis. The number of copies input for each resulting observation is shown on the X-axis. Axes are in log–log scale. The line of best fit displayed is a log–log line with a slope of 1.038, a y-intercept of − 0.4816 and an R^2^ value of 0.9994

To determine the accuracy of the ddPCR system and its limits of detection a custom set of primers and probes were used to detect an empty lentiviral vector, VSVG, with a known sequence. A serial dilution of this vector was prepared starting from $1 \times 10^{6}$ copies per microliter, diluting down to $1$ copy per microliter using a dilution factor of 10. These dilutions were then analyzed using the ddPCR system to determine if the observed copy numbers would reflect the input concentration. It was found that at concentrations of $1 \times 10^{5}$ and $1 \times 10^{6}$ copies per microliter, the QuantaSoft software reported copy numbers of $1 \times 10^{6}$ copies with large error or would not report values at all. Therefore, inputs of $1 \times 10^{5}$ and $1 \times 10^{6}$ copies per microliter were subsequently considered above the limits of detection. For 5 input concentrations below this limit of detection beginning with approximately 1 × 10^4^ copies per microliter, observed copy numbers were averaged (n = 3) and plotted on a log–log graph. A log–log line of best fit revealed good correlation between input concentration and average observed copy number and a R^2^ value of 0.9907 (Fig. 1a).

To estimate the lower limit of detection (LoD), another VSVG serial dilution was prepared using a greater number of dilutions. The dilution series consisted of a nine-point dilution starting from a concentration of 5 × 10^4^ copies per microliter, with a fivefold dilution factor for each point. The dilution series was analyzed using the QuantaSoft software and results plotted on a log–log graph. There was an average of 20,341.84 droplets per well with a maximum of 21,466 droplets in one well. A log–log line of best fit revealed good correlation between input concentration and average observed copy number with an R^2^ value of 0.9994. The lower limit of detection was estimated to be 0.13 copies per microliter as this data point deviated outside of the 95% confidence interval of the line of best fit with a significant standard error of 0.035 (Fig. 1b).

**Fig. 2:**
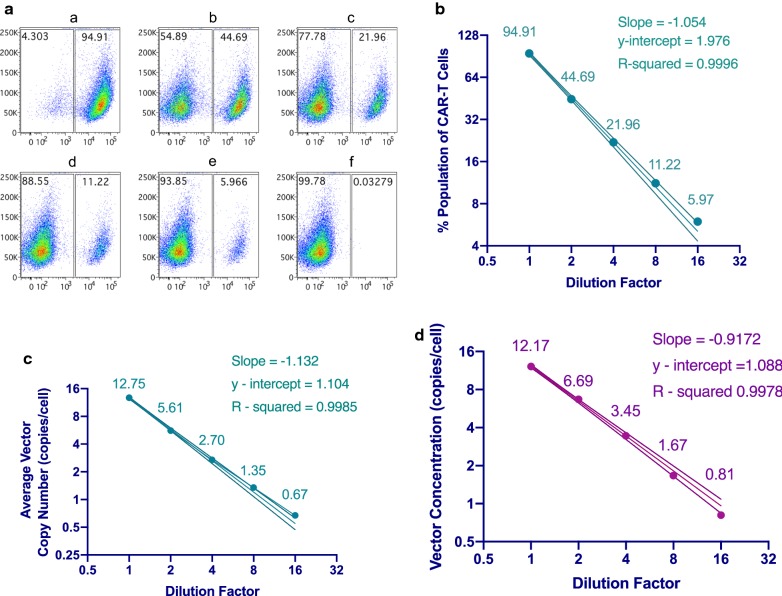
Analysis of Assay Dynamic Range using Transduced T cells and Lentiviral Vector. T cells engineered to express an HPV-16 E7 oncogene-specific TCR and the lentiviral vector used to produce these cells were used to assess the dynamic range of the ddPCR vector copy number assay. **a** HPV16 E7 TCR engineered T cells were serial diluted twofold with untransduced lymphocytes and percentage of TCR-beta positive cells was determined by flow cytometry. HPV16 E7 TCR engineered cells that were not diluted are shown in panel a, cells diluted 1:2 are shown in panel b, 1:4 diluted in panel c, 1:8 diluted cells in panel d, and 1:16 diluted in panel e. Flow cytometry analysis of untransduced lymphocytes are show in panel f. The representative flow plots were gated on viable CD3^+^ T cells. **b** For each of the HPV16 E7 TCR engineered T cell samples serially diluted with untransduced lymphocytes, the log of the transduction efficiency and the log of the dilution factor is shown. The line of best fit with confidence intervals are shown. Each point represents the average of two measurements. **c** The HPV16 E7 TCR engineered T cell samples were also analyzed for vector copy number by ddPCR and the log of the vector copy number and dilution for each sample is shown. Each point represents the average of two measurements. The line of best fit with confidence intervals is shown. **d** The HPV-16 E7 TCR lentiviral vector was diluted with DNA from untransduced lymphocytes cells and evaluated by ddPCR and the log of the vector copy number and dilution for each sample is shown. Each point represents the average of two measurement. The line of best fit with confidence intervals is shown

The assay dynamic range was also tested with T cells engineered to express a TCR specific for HPV16 E7 oncoprotein using a retroviral vector. HPV16 E7 oncogene TCR engineered T cells with a 94.9% transduction efficiency were diluted with untransduced T cells serially by twofold to a 1:16 dilution and the proportion of TCR engineered T cells in each aliquot was evaluated by flow cytometry (Fig. 2a). The diluted TCR engineered cells were tested in duplicate and when plotted on a log–log graph there was a linear relationship between the transduction efficiency measured and the fold dilution (Fig. 2b). Each of these aliquots was also evaluated for vector copy number using the ddPCR assay. The undiluted cell sample contained 12.75 vector copies per cell and when plotted on a log–log graph there was a linear relationship between the measured vector copy number in the T cell samples and the dilution factor (Fig. 2c). The assay was also tested by diluting the HPV16 E7 oncoprotein TCR retroviral vector with DNA from untransduced cells serially by a factor of 2 and for this dilution method there was also a linear relationship between the vector copy number and the dilution factor (Fig. 2d). This demonstrates the fidelity of the ddPCR system, as a reduction by half of amount of DNA from CAR T cells in a sample produces a reduction by half of the observed average copy number.

### Assessment of the consistency ddPCR measurement of CAR vector copy number

#### Assessing variation across time

**Fig. 3:**
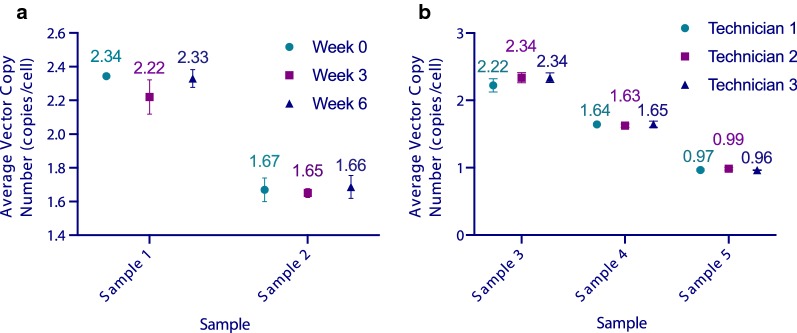
Assessment of Consistency of Vector Copy Number Measured by ddPCR. Anti-BCMA-CAR T cell vector copy number was assessed by ddPCR and assay consistency was assessed over time and among laboratory staff performing the assay. **a** Two different anti-BCMA-CAR T cell samples were tested by the same person prior to cryopreservation and after 3 weeks and 6 weeks of storage at -80 °C. Sample 1 had a mean vector copy number of 2.30 ± 0.08 copies/cell with a CV of 0.04 and Sample 2 had a mean of 1.67 ± 0.05 copies/cell with CV of 0.03. The P-value of a Two-way ANOVA analysis comparing vector copy numbers observed across time was 0.11. **b** Three different anti-BCMA-CAR T samples (fresh or cryopreserved) were tested by 3 different staff working in the same laboratory. Sample 1 had a mean of 2.30 ± 0.09 copies/cell with a CV of 0.04, Sample 2 had a mean of 1.64 ± 0.03 copies/cell with a CV of 0.02, and Sample 3 had a mean of 0.97 ± 0.02 copies/cell with a CV of 0.02. The P-value of a two-way ANOVA analysis comparing the vector copy numbers observed by each technician was 0.22

To evaluate variation in measuring vector copy number across time, two aliquots (sample 1, sample 2) of cryopreserved anti-BCMA-CAR T cell products that were genetically engineered with a retroviral vector were thawed and evaluated, in triplicate, at three different time points (week 0, 3, 6) spaced 3 weeks apart. The same person performed all tests using the same instrument. Two-way ANOVA analysis comparing the vector copy number observed across samples and across time found no significant difference between time points (P > 0.05) (Fig. 3a). This shows that the time that a sample is thawed and analyzed has minimal impact on the measured vector copy number.

#### Assessing Variation Among Assay Laboratory Staff

To assess variation in measuring vector copy number across individuals performing the assay independently, three technicians set up, ran the instrument and analyzed the same aliquots obtained from three CAR T cell products. A two-way ANOVA analysis found no significant differences between copy numbers observed across technicians (P > 0.05) (Fig. 3b), highlighting the repeatability and robustness of this assay.

### Application of ddPCR in CAR T cell manufacturing

#### Transduction conditions and vector copy number

**Fig. 4:**
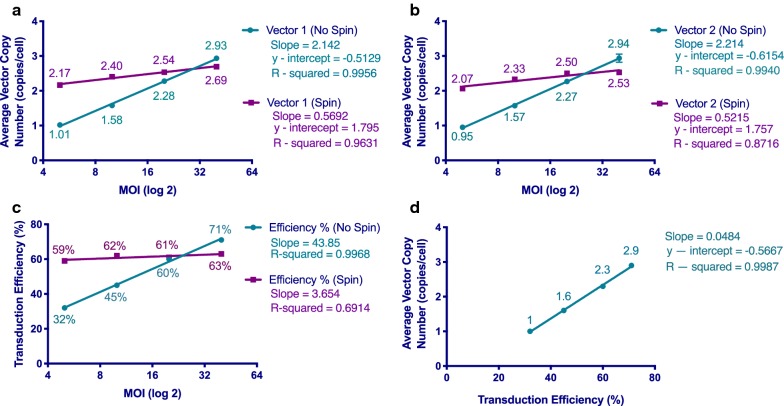
Demonstration of the Application of ddPCR in CAR T Cell Manufacturing. Vector copy number was measured in anti-GPC3-CAR T cells using different vector MOIs and spin transduction centrifuge speeds. Two different regions were assessed. **a** Average vector copy number measured in CAR T product produced using primers specific to the EF1a promoter region, plotted against MOI on a semi-log plot with a log-2 scaled X-axis. Data points are means of triplicates grouped by centrifugation condition. **b** Average vector copy numbers measured in CAR T cell products produced using primers against the scFv CAR region, plotted against MOI on a semi-log plot with a log-2 scaled axis. Data points are means of triplicates grouped by centrifugation condition. Centrifugation was observed to increase resultant average copy number at lower MOI, decreasing the slope of the semi-log lines. **c** Transduction efficiency was measured by flow cytometry for anti-GPC3-CAR T cells products and was plotted against MOI on a semi-log plot with a log-2 scaled X-axis. Values plotted are single data points observed in a CAR T cell product processed under two different conditions. **d** For each anti-GPC3-CAR T cell sample, vector copy number was plotted against transduction efficiency with linear fit. The data plotted was from a single anti-GPC3-CAR T product

We used the ddPCR vector copy assay to analyze the consistency of the assay when targeting different regions within an anti-GCP3-CAR lentiviral vector. Two different regions were targeted, the EF1a promoter region and the scFv CAR region. We showed that there was no significant difference in copy number at multiple multiplicity of infections (MOI) when comparing results from targeting either of these two regions (Figure A). Data obtained from the ddPCR system also allowed for some preliminary analysis of CAR T cell production and how manufacturing conditions may impact resulting vector copy number. We used the ddPCR vector copy assay to analyze the effects of differences in multiplicity of infection (MOI) and centrifugation conditions (spinoculation) during transduction on CAR T cells. The MOIs used for transfection were 5, 10, 20, and 40. The centrifugation conditions applied were centrifugation at 1000 G at 32 °C for 2 h or no centrifugation at all. Furthermore, independent of the region assayed, it was found that greater centrifugation forces increased the resulting copy number at the lower range of MOI’s evaluated but not at the higher MOI’s (Fig. 4a, b). We also evaluated the effect of MOI and centrifuge speed on transduction efficiency measured by flow cytometry for CAR T cells manufactured using a lentiviral vector and the results were similar (Fig. 4c). The relationship between transduction efficiency and vector copy number for CAR T cell samples was examined and it was observed that there was a strong correlation between transduction efficiency and vector copy number (Fig. 4d).

## Discussion

These results show that ddPCR can reliably measure the average number of vector copies integrated into CAR and TCR engineered T cells and the system that we tested can provide precise, replicable results. Measurements of vector copy number remained stable across varying input concentrations, time-points, technicians, and laboratory settings. The practical implications of these results are that this ddPCR system can accommodate a range of DNA inputs.

Within a laboratory, the results were consistent even with different staff preforming the assay. As copy number measurements remained consistent across technicians, the ddPCR system demonstrated tolerance for different users within a controlled environment. A point of consideration is that the droplet generation step of the ddPCR protocol used is automated, reducing human error.

We are currently using this method to measure vector copy number in several clinical CAR T cell and TCR engineer T cell therapies. Several other types of genetically engineered somatic cells for clinical use are being produced from autologous blood cells. Autologous genetically engineered CD34+ cells are used to treat patients with inherited monogenetic immune deficiencies such as chronic granulomatous disease and severe combined immune deficiency [24, 25]. They are also being used to treat hemoglobinopathies including sickle cell disease and beta thalassemia [26, 27]. In addition to the CAR and TCR engineered T cells, genetically engineered NK cells are being used as cancer immunotherapies [28]. It is expected that this ddPCR assay will be effective in measuring vector copy number in all of these genetically engineered cells.

One limitation of this assay is that it measures the average number of vector copies integrated into the entire population of cells tested. When vector copy number measurements are being used to as a safety measure to ensure that clinical cell therapies do not exceed a defined threshold for the number of copies of vector inserted into the cells, it may be better to measure vector copy number in only transduced cells. However, vector transduction efficiency measured by flow cytometry can be used along with the average vector copy number for the entire cell population to estimate the average vector copy number of transduced cells.

## Conclusion

We show that ddPCR is an effective method to assay vector copy number in CAR T cells. The assays should also be applicable to other types of engineered T cells and hematopoietic stem cells.

## Data Availability

Not applicable.
